# Supplementary material for: Targeting ADAR stability: a novel approach to combat glioblastoma
Source: Front Oncol. 2026 Aug 6;16:1827793. doi: 10.3389/fonc.2026.1827793 (PMC13489870; doi:10.3389/fonc.2026.1827793)
Supplement: Supplementary file 1 [file SupplementaryFile1.zip › Supplementary file 1/D_Arginine_and_D_ornithine_metabolism.pdf]

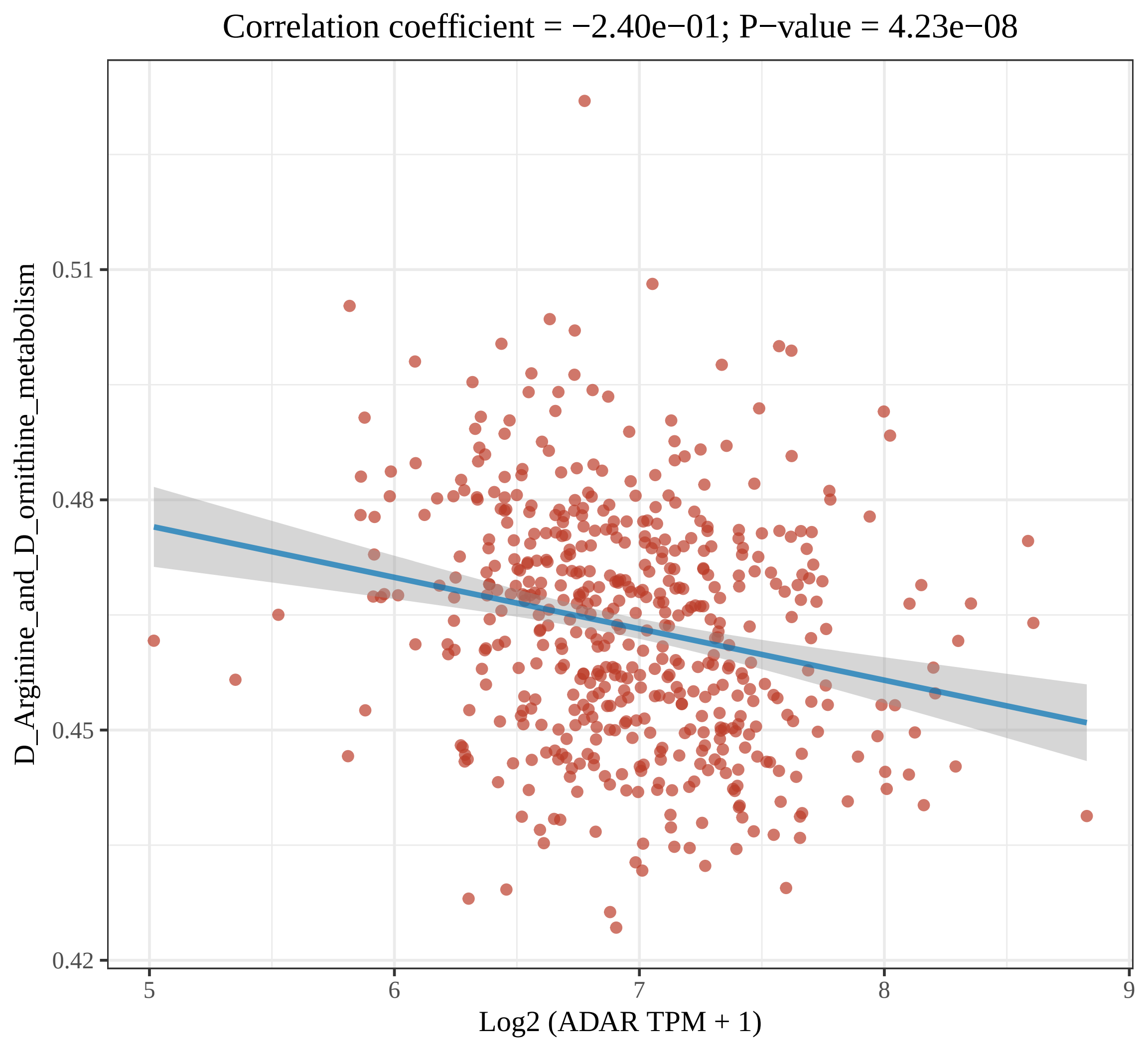
Correlation coefficient = −2.40e−01; P−value = 4.23e−08
D_Arginine_and_D_ornithine_metabolism
0.51
0.48
0.45
0.42
5
6
7
8
9
Log2 (ADAR TPM + 1)
